# Fatty Acid Composition, Molecular Species, and Ameliorating Effects of Tilapia Head Phospholipids on High-Fat Diet-Induced Metabolic Syndrome in Mice

**DOI:** 10.3390/nu18183076

**Published:** 2026-09-20

**Authors:** Xia Gao, Hui Yu, Xiangzhou Yi, Shuxin Gao, Xuanri Shen

**Affiliations:** 1 School of Food Science and Engineering, Hainan Tropic Ocean University, Sanya 572022, China; gaoxia@hntou.edu.cn (X.G.); xiangzhouyi1995@hainanu.edu.cn (X.Y.); 22210832000029@hainanu.edu.cn (S.G.); 2 School of Food Science and Engineering, Hainan University, Haikou 570228, China; yuhui19970607@163.com

**Keywords:** phospholipids, metabolic syndrome, gut microbiota

## Abstract

Background/Objectives: The fish head contains a large number of bioactive lipids for preparing functional foods. In this study, we analyzed the fatty acid components and molecular species of tilapia head phospholipids (TH-PL), and investigated the preventive effect of them on metabolic syndrome (MS). Methods: Twenty-three fatty acids were identified in TH-PL, and the percentage of n-3 PUFA and n-6 PUFA was high, occupying 48.21% of total fatty acids. And sixty-two phospholipids (PL) molecular species belonging to seven types of PL were identified using UPLC-Q-TOF-MS. The contents of the various types of TH-PL were generally in the order PC > PE > SM > PS > LPC > PI > LPE. Among them, PC (16:0/18:1), PE (P-16:0/22:4), PS (18:0/22:5), LPC (16:0), PI (16:0/20:4), LPE (18:0), and SM (d16:0/18:1) were the major molecular species of TH-PL. Moreover, animal experiments were conducted to evaluate the preventive effect of TH-PL on high-fat diet (HFD)-induced MS mice. Results: Animal experiments revealed that TH-PL could significantly inhibit obesity, improve insulin resistance and dyslipidemia in high-fat diet (HFD)-induced MS mice. Intake of TH-PL could also block lipid synthesis in the liver by modulating the expression of lipogenesis-related genes and proteins, such as up-regulation of AMPK and down-regulation of PPARγ, SREBP-1c, and FAS. In addition, TH-PL could relieve MS directly and indirectly by counteracting gut microbiota disturbance caused by HFD. Conclusions: These data suggested that it may be a good choice to develop new functional foods containing TH-PL for dietary intervention in MS.

## 1. Introduction

With the improvement of living standards and changes in diet structure, the exorbitant ingestion of high-calorie and high-fat foods has led to a sharp increase in the incidence of metabolic syndrome (MS) [1]. MS is considered to be a combination of abdominal obesity, lipid metabolism disorders, insulin resistance (IR), hyperlipidemia and other pathological phenomena, which are closely linked to the occurrence of various chronic diseases, such as obesity, diabetes and non-alcoholic fatty liver. The pathogenesis of MS is complex and may be partly attributed to environmental, genetic and dietary factors [2]. However, accumulating evidence indicates that gut microbiota disturbance induced by a high-fat diet (HFD) is also a key factor in the development of obesity, fatty liver and other MS [3]. The disrupted gut microbiota directly damage the intestinal barrier and then further aggravate lipid metabolism disorder by triggering inflammation or oxidative stress [4]. Therefore, MS seriously threatens human health, which makes it a global public health problem that urgently needs to be resolved. Current treatment approaches for MS include medication, dietary management, and exercise therapy. Among these, dietary nutritional interventions offer a safer profile than pharmacotherapy and are increasingly becoming a vital component in the adjunctive treatment and prevention of chronic diseases. In particular, functional foods from natural sources provide many health benefits with few side effects, which makes them a potential choice for the prevention and therapy of MS. Phospholipids (PLs) are amphipathic lipid compounds with phosphate groups, which are critical components of cellular membranes. Based on their linker, PLs are classified into phosphatidylcholine (PC), phosphatidylethanolamine (PE), phosphatidylserine (PS), phosphatidylinositol (PI), lysophosphatidylcholine (LPC), lysophosphatidylethanolamine (LPE), phosphatidic acid (PA) and sphingomyelin (SM). As a multi-purpose biological macromolecule, exogenous PLs have been confirmed by many pharmacological studies as a new type of dietary supplement and functional food due to their excellent lipid-lowering, anti-inflammatory and anti-cancer activities [5,6].

PLs derived from different origins exhibit substantial structural heterogeneity, which can be attributed to variations in their polar headgroups, fatty alkyl chain lengths, and saturation levels. The diverse molecular species and structures result in different biological activities. Compared to PLs derived from terrestrial animals and plants, those from fish are valued for their abundance of *n*-3 polyunsaturated (PUFA), particularly eicosapentaenoic acid (EPA) and docosahexaenoic acid (DHA), which are essential to human health, thereby conferring superior nutritional value [7,8]. Thus, fish-derived PLs demonstrate the twofold functions of PLs and *n*-3 PUFA. Shirouchi et al. found that salmon roe PLs could inhibit fatty acid synthesis in rats and exhibited a better adiposity effect than soybean phospholipids (S-PLs) [9]. Similarly, cuttlefish spawn PLs were proven to be more efficient in ameliorating lipid metabolism disorder than S-PLs and egg yolk PLs, which shows the great advantages of PLs from aquatic organisms [10].

In recent years, the fishery processing industry has experienced rapid development. However, a significant portion of fish heads generated during processing are discarded as waste, leading to both resource wastage and environmental pollution. Notably, tilapia, which is widely cultivated worldwide, contains abundant PLs in its heads, which makes it a potential raw material for value-added aquatic health products [11]. So far, there have been few reports on the lipid composition and biological activity of tilapia head phospholipids (TH-PLs). Therefore, the aim of this study was to characterize the fatty acid profile and molecular species of TH-PLs and to evaluate their ameliorative effects on MS in an HFD mouse model, along with exploring the potential underlying mechanisms. Here, we extracted TH-PLs and then identified the fatty acids composition and molecular species of TH-PLs by GC-MS and UPLC-Q-Orbitrap HRMS, respectively. In addition, the ameliorative effect of TH-PLs on MS and its potential mechanism were comprehensively evaluated. Collectively, the structural elucidation and in vivo functional evaluation of TH-PLs provide a rationale for their targeted deployment in future interventions.

## 2. Materials and Methods

### 2.1. Chemicals and Reagents

Tilapia heads were obtained from Hainan Xiangtai Fisheries (Hainan, China). The HFD was purchased from Hunan Slac Jingda Laboratory Animal Co., Ltd. (Changsha, China). S-PLs were purchased from Shanghai Enzyme-linked Biotechnology Limited Company (Shanghai, China). All other chemicals were acquired from Guangzhou Chemical Reagent Factory (Guangzhou, China).

### 2.2. Extraction of TH-PLs

TH-PLs were isolated following the method described by [12], with ultrasonic-assisted extraction applied to the fish heads. Briefly, the fish heads were ground and sonicated in 95% (*v*/*v*) ethanol at the ratio of 1:10 (tilapia head: ethanol) (*m*/*v*) for 1.5 h three times. After filtration, the extracts were concentrated by rotary evaporation at 45 °C; mixed with a solution containing chloroform, methanol, and water (8:4:3, *v*/*v*/*v*); and centrifuged at 4000× *g* rpm at 25 °C for 10 min. The lower organic phase was collected, mixed with 0.2 volume of 0.9% (*w*/*v*) NaCl and vortex mixed for 1 min, then centrifuged at 4000× *g* rpm at 25 °C for 10 min to obtain the lower organic layer and concentrated by rotary evaporation. A silica gel column chromatography system was used to separate the concentrate by medium-pressure purification. Extract constituents were eluted with chloroform, acetone, and methanol in sequence. The TH-PLs were collected from methanol eluent, which was evaporated and dried under N_2_ flow. The prepared TH-PLs were stored at −20 °C until use.

### 2.3. Fatty Acid Analysis of TH-PLs

The TH-PLs were saponified with 0.5 M methanolic NaOH, and fatty acid methyl esters (FAMEs) were extracted with *n*-hexane. Then, the FAMEs were analyzed using an Agilent 8890B-5977B GC/MSD equipped with a DB-Fast FAME column (20 m × 0.18 mm, 0.2 μm).

### 2.4. Molecular Species Analysis of TH-PLs

TH-PL molecular species were analyzed using ultra-high-performance liquid chromatography–quadrupole electrostatic field orbitrap high-resolution mass spectrometry (UPLC-Q-Orbitrap HRMS, Thermo Fisher Scientific, Waltham, MA, USA). The UHPLC Dionex Ultimate 3000 (Thermo Fisher Scientific, Waltham, MA, USA) equipped with an ACQUITY UPLC CSH C18 column (2.1 × 150 mm, 1.7 μm, Waters, Milford, MA, USA) was used for chromatographic separation. Mass spectrometry was conducted in both positive and negative ionization modes. Data acquisition employed a data-dependent scan (FS-ddMS2).

### 2.5. Animals and Treatment

All animal experiments were conducted in accordance with the Guidelines for the Care and Use of Laboratory Animals of Hainan University and approved by its Animal Ethics Committee (No. HNUAUCC-2021-00046). Male C57BL/6 mice (20.0 ± 2.0 g) were obtained from TianQin Biotechnology Company (Changsha, China; License ID: SCXK2019-0014). The animals were housed under controlled conditions with free access to food and water. The mice were randomly divided into four groups (*n* = 8): Control (normal diet), Model (HFD), S-PL (HFD + 200 mg/kg S-PL), and TH-PL (HFD + 200 mg/kg TH-PL). Treatments were administered orally (the figure in Section 3.3). The HFD and TH-PL diets were isocalorically matched. The experiment lasted eight weeks, with weekly recordings of body weight and body length. During the 8-week experiment, some samples were excluded from specific assays due to hemolysis, insufficient tissue, or technical errors during processing. The exact sample size (*n*) for each measurement is indicated in the corresponding figure legends.

At the end of the treatment period, mice were anesthetized with pentobarbital and euthanized. Blood was collected from the orbital vessels. Liver tissues were excised, photographed, and weighed; the left lobe was preserved for histopathological analysis. Cecal contents were also collected and stored at −80 °C for further analysis. Lee’s index, liver index, and epididymal fat index were calculated according to the following formulas:

(1) $${\mathrm{Lee}}^{\prime }s\;\mathrm{index}\;=\;[{\mathrm{body}\;\mathrm{weight}\;\left(g\right)}^{\frac{1}{3}}/\mathrm{body}\;\mathrm{length}\;\left(\mathrm{cm}\right)]\;\text{×}\;1000$$

(2) $$\mathrm{Liver}\;\mathrm{index}=\mathrm{liver}\;\mathrm{weight}\;\left(g\right)/\mathrm{body}\;\mathrm{weight}\;\left(g\right)\;\text{×}\;100\%$$

(3) $$\mathrm{Epididymal}\;\mathrm{fat}\;\mathrm{index}=\mathrm{epididymal}\;\mathrm{fat}\;\mathrm{weight}\;\left(g\right)/\mathrm{body}\;\mathrm{weight}\;\left(g\right)\;\text{×}\;100\%$$

### 2.6. Determination of IR Index

Fasting serum glucose and insulin levels were measured using commercial blood glucose test strips and an ultrasensitive mouse insulin ELISA kit, respectively. IR was assessed via the homeostasis model assessment (HOMA-IR) using Formula (4):

(4) $$\text{HOMA-IR}\;\mathrm{index}\;=\;\left[\mathrm{fasting}\;\mathrm{glucose}\;,\left(\mathrm{mmol}/L\right),\;\text{×}\;\mathrm{fasting}\;\mathrm{insulin}\;,\left(\mu U/\mathrm{mL}\right)\right]/22.5$$

### 2.7. Serum and Hepatic Biochemical Assay

The concentrations of Triglycerides (TG), Total cholesterol (TC), High-density lipoprotein cholesterol (HDL-C), Low-density lipoprotein cholesterol (LDL-C), Alanine Aminotransferase (ALT), Aspartate Aminotransferase (AST), Adiponectin (ADPN), Leptin (LEP) and free fatty acids (FFAs) in samples were determined using commercially available kits. Liver tissues from 3 randomly selected mice per group were used for Western blot analysis.

### 2.8. Hematoxylin and Eosin (H&E) Staining and Oil Red O Staining

Liver tissues were fixed in 4% formalin, sectioned (4 μm), and stained with H&E. For Oil Red O staining, tissues were fixed in 4% paraformaldehyde, dehydrated in sucrose solution and stained with Oil Red O. All sections were observed at 400× magnification using microscope.

### 2.9. Gut Microbiota Analysis

Gut microbiota diversity and composition were analyzed by high-throughput sequencing. Total genomic DNA was extracted from cecal contents, and the V3–V4 region of the 16S rRNA gene was amplified by PCR, followed by quantification and purification of the products. Sequencing was performed on an Illumina HiSeq2500 platform (Illumina, San Diego, CA, USA), and data were analyzed using the Majorbio Cloud Platform. The 16S rDNA sequences were deposited in the NCBI database under accession number PRJNA803811.

### 2.10. Quantification of Short-Chain Fatty Acids (SCFAs)

Cecal content samples were pretreated following the method of Zhuang, et al. [13], with minor modifications. Quantitative analysis was performed using an Agilent 8890B-5977B GC-MS (Agilent, St. Clara, CA, USA) equipped with a flame ionization detector and an HP-FFAP capillary column.

### 2.11. Quantitative Real-Time Polymerase Chain Reaction (qPCR)

Total RNA was extracted from mice liver using the Eastep Super Total RNA Extraction Kit (Promega, Shanghai, China) and reverse-transcribed into cDNA using the RevertAid First Strand cDNA Synthesis Kit (Thermo Fisher Scientific, Waltham, MA, USA). Quantitative PCR amplification was performed with SuperReal PreMix Plus (SYBR Green) (TIANGEN Biotech, Beijing, China) using gene-specific primers (Table 1). Relative gene expression was normalized to GAPDH and calculated accordingly.

### 2.12. Western Blotting (WB)

Liver proteins were extracted by homogenization and centrifugation, separated via SDS-PAGE, and transferred onto PVDF membranes. The membranes were blocked with 5% skim milk and incubated with primary antibodies against AMPK, phospho-AMPK, PPARγ, FAS, and SREBP-1c. After washing, membranes were incubated with HRP-conjugated secondary antibodies. Protein bands were visualized using an ECL chemiluminescence kit and captured with a Tanon-5200 imaging system. Densitometric analysis was performed using ImageJ v1.52a, with β-actin serving as the loading control.

### 2.13. Statistical Analysis

Data are expressed as mean ± SEM. Statistical comparisons among multiple groups were performed by one-way analysis of variance (ANOVA) followed by Tukey’s post hoc test. For all analyses, *p* < 0.05 and *p* < 0.01 were considered to indicate significant and extremely significant differences, respectively.

## 3. Results

### 3.1. Fatty Acid Composition of the TH-PLs

A total of twenty-three fatty acids with carbon chain lengths ranging from C14 to C24 were identified in the TH-PLs (Table 2). The overall composition was categorized into saturated (SFA, 32.59%), monounsaturated (MUFA, 19.20%), and PUFA (48.21%) fatty acids. The SFA fraction was predominantly composed of C16:0 (19.33%) and C18:0 (10.69%), while C18:1 (17.25%) was the major constituent of the MUFA fraction. The PUFA composition of the TH-PLs was characterized by a high proportion of *n*-6 (30.30%) and *n*-3 (17.91%) fatty acids. Additionally, the C20 content significantly exceeded that of C18, which aligns with existing studies on animal fatty acids. Notably, the widely recognized functional fatty acids, EPA and DHA, were identified in TH-PLs at contents of 0.42% and 6.78%, respectively.

### 3.2. Molecular Species of TH-PLs

**Figure 1:**
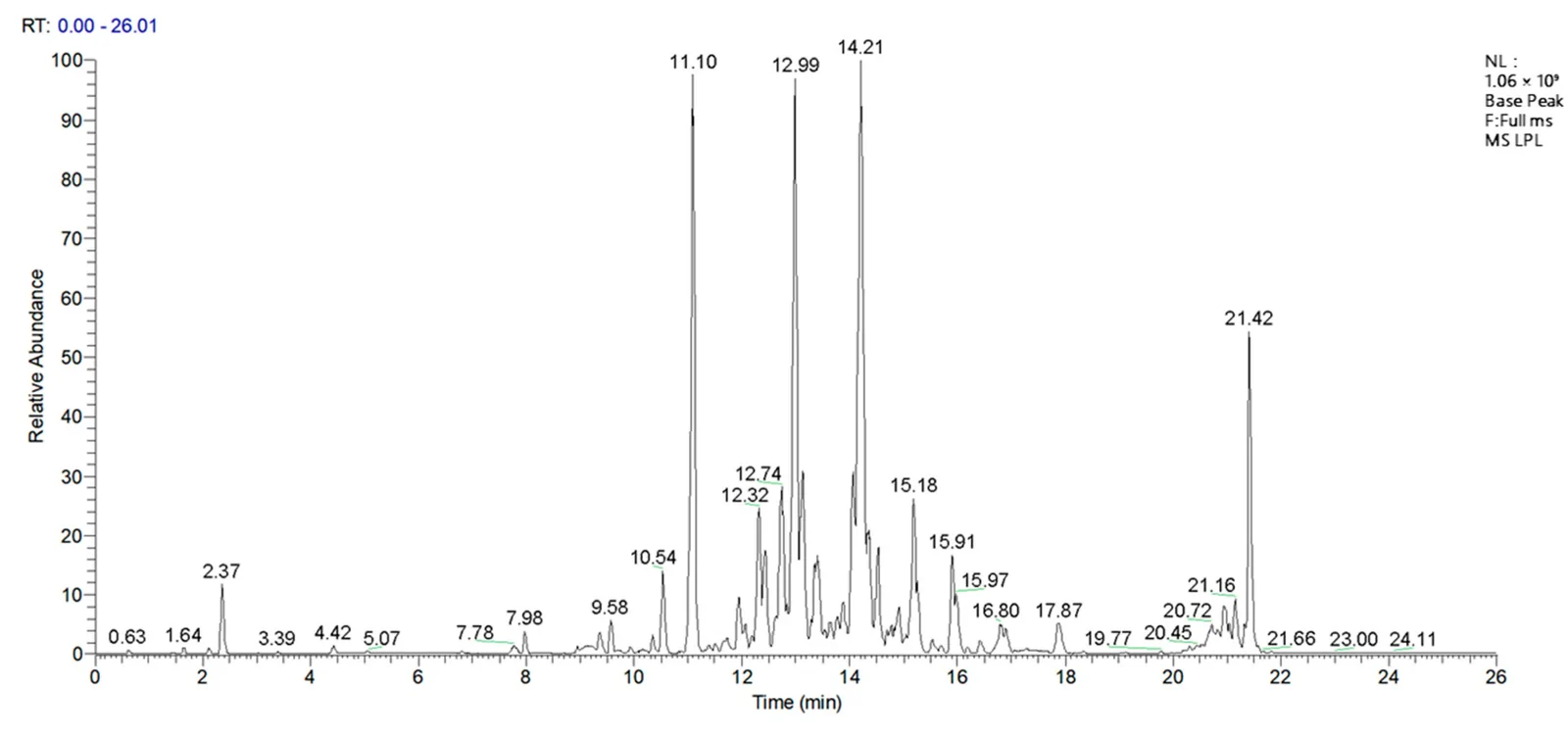
Total ion flow diagram of TH-PLs.

Non-targeted lipidomic profiling of the TH-PLs was performed using UPLC-Q-Orbitrap HRMS. Seven principal PL subclasses were mainly detected, including PC, PE, PS, LPC, PI, LPE and SM, with fatty acyl chains ranging from 16 to 26 carbon atoms and containing zero to six double bonds per chain. PC is the major subclass (55.62%), and PE accounted for 18.01% of the TH-PLs. SM represented a moderate proportion (5.79%), while PS was present at a lower level (1.57%). The contents of LPC, PI, and LPE were relatively minor (0.39%, 0.23%, and 0.12%, respectively) (Table 3). These findings are consistent with previous reports [14]. A total of 62 distinct PL molecular species were identified, comprising 20 PC, 15 PE, seven PS, five LPC, five PI, three LPE, and seven SM species. The total ion chromatogram (TIC) of the TH-PLs is presented in Figure 1, and Table 4 summarizes the molecular ions detected for the seven PL classes, including observed mass, molecular formula, retention time (RT), and relative abundance. The relative quantification of individual PL molecular species was based on a comparison of their total peak areas. In the PC subclass, the major molecular species included PC (16:0/18:1), PC (16:0/18:2), PC (18:1/18:2), and PC (18:1/18:1). Within the PE subclass, the predominant species were P-16:0/22:4, 18:1/20:4, and P- 18:1/22:4, with relative abundances of 11.77%, 7.80%, and 6.80%, respectively. The PS subclass was notably enriched in MUFA species such as PS (18:0/22:5), PS (18:0/22:4), and PS (18:0/22:6). The most abundant LPC species were LPC (16:0) and LPC (18:1), with relative abundances of 33.83% and 23.62%, respectively. PI (16:0/20:4) was the dominant PI species, constituting 66.28% of its subclass. The identified LPE species included LPE (18:0) at 50.66%, LPE (20:4) at 26.87%, and LPE (22:6) at 22.45%. In the SM subclass, SM (d16:0/18:1) was the most abundant species (19.17%), followed by SM (d18:1/24:1) (17.97%).

### 3.3. Effect of TH-PL on Growth Parameters in Mice

**Figure 2:**
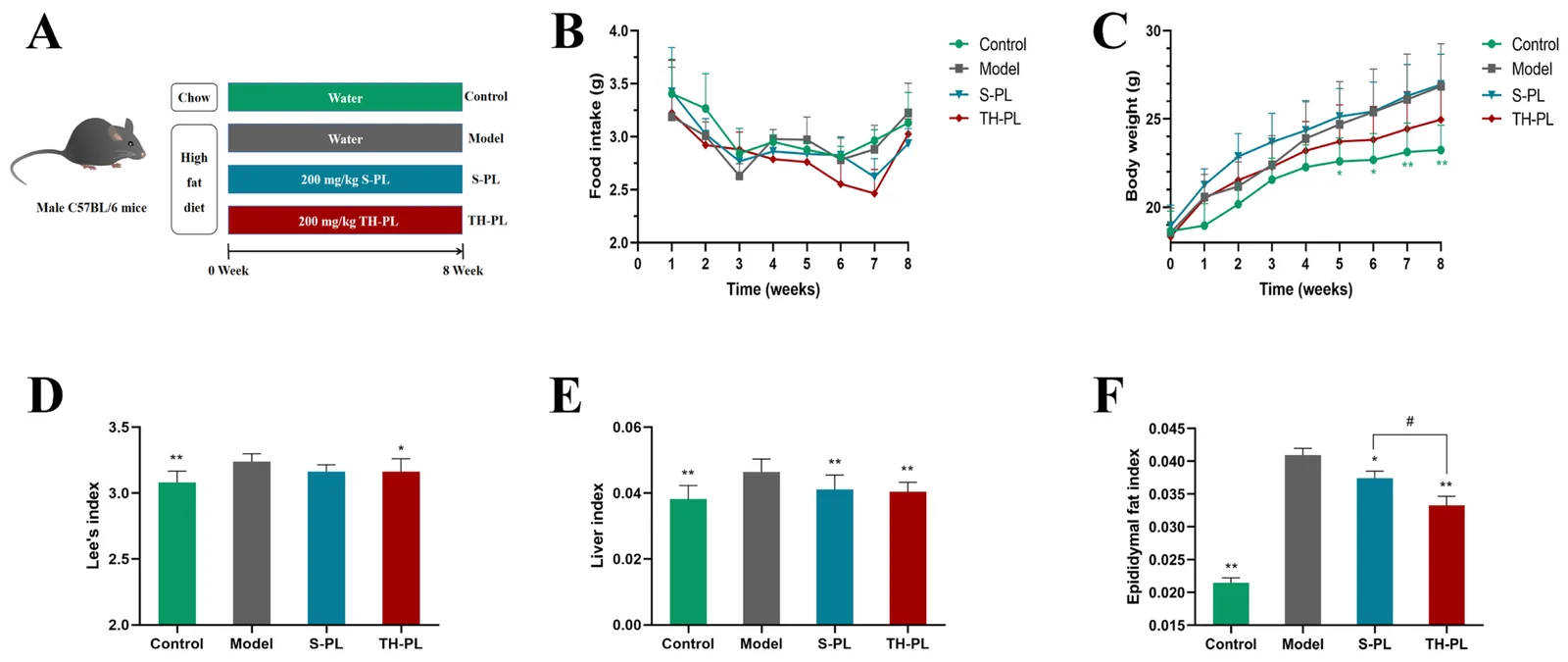
Effect of TH-PLs on growth parameters in mice. (**A**) Schematic diagram of experimental design; (**B**) food intake; (**C**) body weight; (**D**) Lee’s index; (**E**) the liver index; and (**F**) the epididymal fat index. Values were expressed as mean ± SEM in each group. * *p* < 0.05 as compared to the Model group; ** *p* < 0.01 as compared to the Model group. # *p* < 0.05 as compared to the S-PL group (*n* = 6).

As shown in Figure 2B, no remarkable differences in food intake were observed among the Control, Model, S-PL and TH-PL groups. The body weights of the mice in all groups gradually increased over the experimental period (Figure 2C). However, weekly weight gain differed across the treated groups. The Model group exhibited a higher rate of weight gain compared to the other groups, culminating in a final mean body weight that was significantly greater than that of the Control group (*p* < 0.01). By contrast, the final mean body weights of the S-PL and TH-PL groups were slightly lower than that of the Model group, although the differences did not reach statistical significance. The Lee’s index was identified as an indicator of adiposity, as shown in Figure 2D. At the end of the experiment, TH-PL treatment significantly reduced the Lee’s index in MS mice compared with the Model group. The effects of S-PLs and TH-PLs on liver index are presented in Figure 2E. An HFD markedly increased the liver index by approximately 21.64% relative to the Control group, while both the S-PL and TH-PL treatments effectively attenuated this increase. Furthermore, as summarized in Figure 2F, TH-PL treatment significantly decreased the index of epididymal fat, demonstrating a more pronounced anti-adiposity effect than S-PLs (*p* < 0.05).

### 3.4. Effect of TH-PLs on Serum Lipid Level in Mice

**Figure 3:**
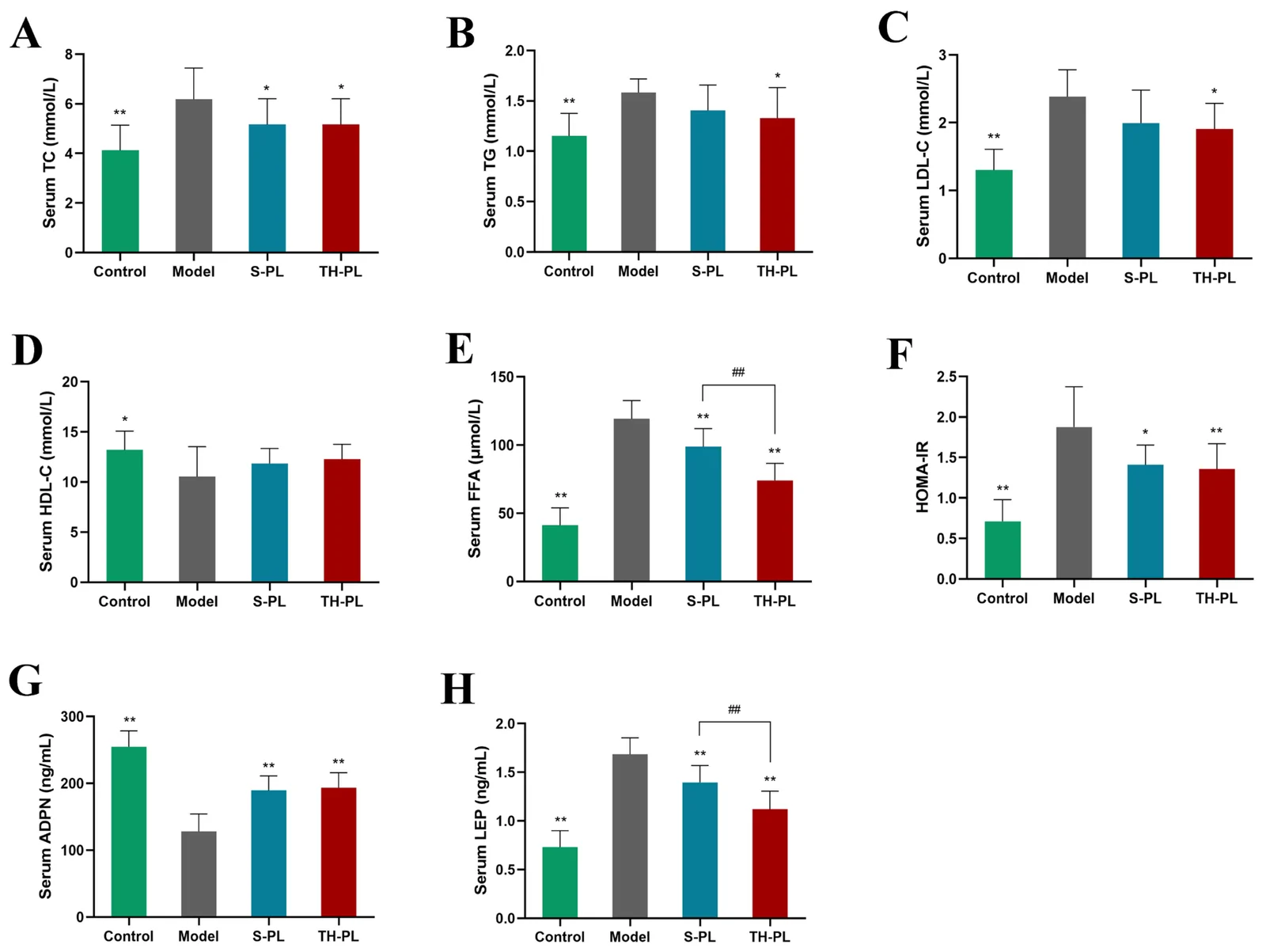
Effect of TH-PLs on serum biochemical indexes in mice. (**A**) Serum TC; (**B**) serum TG; (**C**) serum LDL-C; (**D**) serum HDL-C; (**E**) serum FFA; (**F**) HOMA-IR; (**G**) serum ADPN; and (**H**) serum LEP. Values were expressed as mean ± SEM in each group. * *p* < 0.05 as compared to the Model group; ** *p* < 0.01 as compared to the Model group. ## *p* < 0.01 as compared to the S-PL group (*n* = 6).

Serum lipid levels serve as a biochemical reflection of the dynamic balance between energy intake and expenditure. An HFD readily disrupts this equilibrium, leading to aberrant serum lipid profiles. After HFD feeding, mice in the Model group developed dyslipidemia, characterized by significantly elevated levels of TC, TG, and LDL-C, along with a marked reduction in HDL-C, relative to the Control group (Figure 3A–D). Both the S-PL and TH-PL interventions exerted modulatory effects on serum lipids in MS mice. Specifically, compared with the Model group, the TH-PL group exhibited significantly lowered serum levels of TG, TC, and LDL-C, whereas the S-PL group only exhibited a significant reduction in TC. Furthermore, the TH-PL group also exhibited significantly decreased serum free fatty acid (FFA) levels, and this effect was significantly more pronounced than that in the S-PL group (*p* < 0.01), which underscores the superior lipid-lowering efficacy of TH-PLs.

### 3.5. Effect of TH-PLs on IR in Mice

As shown in Figure 3F, both treatments ameliorated the elevated HOMA-IR in MS mice, with TH-PLs exhibiting a more pronounced effect. ADPN, an insulin-sensitizing hormone, displays an inverse correlation with the IR index. Serum ADPN levels were significantly increased in both the S-PL and TH-PL groups compared to the Model group (*p* < 0.01, Figure 3G). LEP regulates glucose and lipid metabolism and can be regarded as a marker of IR. HFD feeding elevated serum LEP by 130.20% relative to the Control group (*p* < 0.01, Figure 3H). By contrast, both the S-PL and TH-PL groups exhibited significantly reduced serum LEP levels compared to the Model group, with TH-PLs demonstrating superior efficacy.

### 3.6. Effect of TH-PLs on Liver Damage in Mice

**Figure 4:**
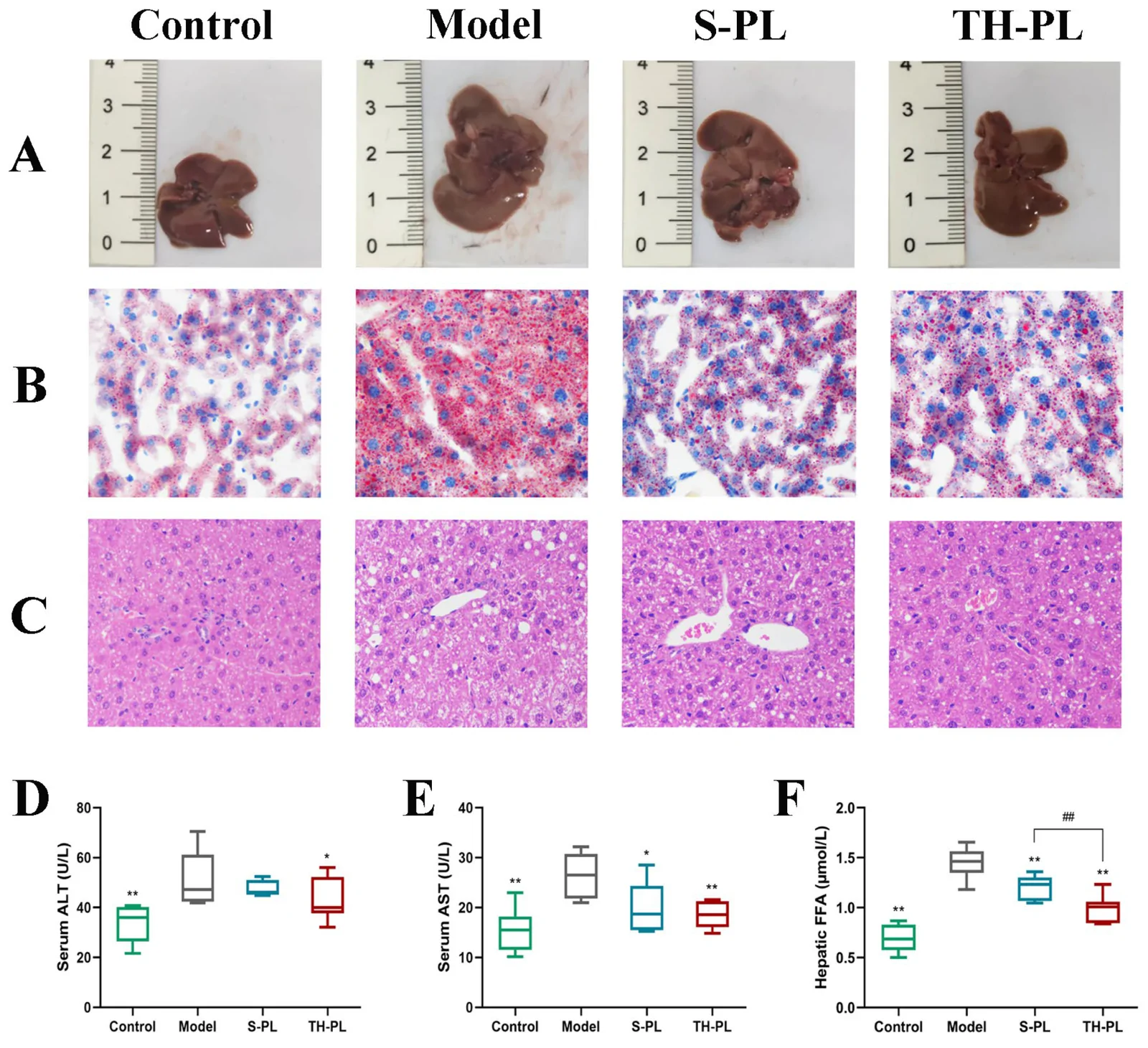
Effect of TH-PLs on liver damage in mice (**A**) Changes in liver morphology; (**B**) Oil red O staining of the liver histology was photographed at 400× magnification, Red indicates lipid droplets and blue indicates nuclei; (**C**) H&E staining of the liver histology was photographed at 400× magnification; (**D**) serum ALT; (**E**) serum AST; and (**F**) FFA level in liver. Values were expressed as mean ± SEM in each group. * *p* < 0.05 as compared to the Model group; ** *p* < 0.01 as compared to the Model group. ## *p* < 0.01 as compared to the S-PL group (*n* = 6).

Through histopathological examination of liver tissues, the extent of liver damage across the four mice groups was evaluated by visual observation (Figure 4A), H&E staining (Figure 4B) and Oil Red O staining (Figure 4C). In the Control group, livers exhibited a dark red color with well-defined margins, normal hepatocyte morphology, and minimal lipid droplet accumulation. By contrast, livers from the Model group showed noticeable hypertrophy, loss of the typical dark red coloration, a disorganized arrangement of hepatocytes, and the presence of extensive fatty vacuoles, which indicates that an HFD likely promoted hepatic lipid accumulation. In the TH-PL group, these pathological alterations were markedly attenuated: liver color and morphology were largely restored to normal, hepatocytes were more tightly arranged, and Oil Red O staining revealed a significant reduction in lipid droplets, with only occasional fatty vacuoles. In addition, in the S-PL group, mild steatosis was observed in some hepatocytes, along with sparse fatty vacuoles within the tissue.

Serum levels of ALT and AST are key biomarkers for assessing hepatic injury, with their concentrations being directly proportional to the extent of hepatocellular damage. As illustrated in Figure 4D,E, mice in the Model group exhibited significant liver dysfunction, characterized by a marked elevation in serum ALT and AST levels compared with the Control group (*p* < 0.05). Treatment with TH-PLs effectively attenuated this increase, reducing ALT and AST activities by 16.99% (*p* < 0.05) and 29.77% (*p* < 0.01), respectively. By contrast, S-PL administration significantly decreased AST levels by 24.51% (*p* < 0.05) but did not lead to a statistically significant reduction in ALT.

The accumulation of excess FFAs in the liver is a key contributor to lipid deposition and hepatocellular injury. As shown in Figure 4F, hepatic FFA levels in the Model group were significantly higher than those in the Control group. Both the TH-PL and S-PL treatments effectively reversed this HFD-induced elevation, resulting in FFA levels that were significantly lower than those in the Model group (*p* < 0.01). Notably, TH-PLs were more effective than S-PLs at reducing hepatic FFA accumulation (*p* < 0.01).

### 3.7. Effect of TH-PLs on Gut Microbiota and Its Metabolites

**Figure 5:**
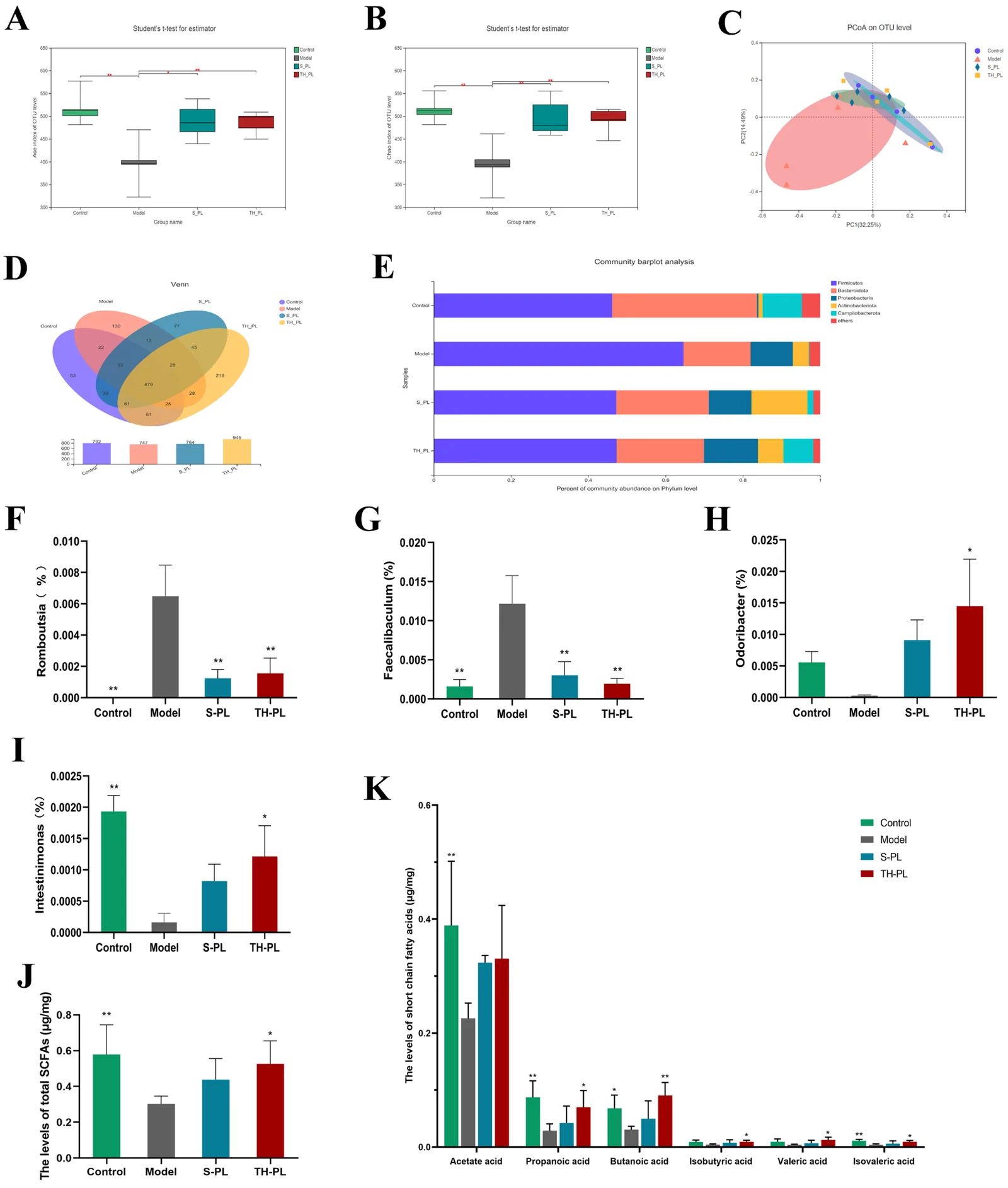
Effect of TH-PLs on gut microbiota and its metabolites. (**A**) ACE index; (**B**) Chao1 index; (**C**) principal co-ordinates (PCoA) analysis based on Unweighted UniFrac distance, he shaded ellipses represent the distribution areas of the samples in each group; (**D**) Venn diagram, the overlapping shaded regions represent the shared and unique OTUs among the different groups; (**E**) microbial community profiling at the phylum level for each group; (**F**) the relative abundance of *Romboutsia*; (**G**) the relative abundance of *Faecalibaculum*; (**H**) the relative abundance of *Odoribacter*; (**I**) the relative abundance of *Intestinimonas*; (**J**) the total levels of SCFAs; and (**K**) the levels of specific SCFAs. Values were expressed as mean ± SEM in each group. * *p* < 0.05 as compared to the Model group; ** *p* < 0.01 as compared to the Model group.

A well-established link exists between MS and gut microbiota dysbiosis. To elucidate the restorative effect of TH-PLs on the gut microbiome in MS mice, high-throughput sequencing of 16S rDNA based on V3-V4 hypervariable regions was used to analyze the variations in the gut microbial structure. The α-diversity indices (ACE and Chao1), which reflect microbial community richness, showed that the HFD reduced gut microbial diversity compared with the Control group (Figure 5A,B). This reduction was significantly restored by both TH-PL and S-PL treatments. Importantly, TH-PLs were superior to S-PLs in reversing the HFD-induced decline in both indices (*p* < 0.01).

The overall structural changes in the gut microbiota communities of the groups were evaluated by unweighted Unifrac-based PCoA at the OTU level. As suggested in Figure 5C, the overall structure of the gut microbiome in the Model group was changed and significantly different from that in the Control group, which may have been caused by the HFD.

Unweighted Unifrac-based PCoA at the OTU-level distances showed that HFD feeding significantly altered the overall structure of the gut microbiota (Figure 5C). While both the TH-PL and S-PL treatments modulated this shift, the TH-PL group’s microbiota composition was notably closer to that of the Control group than to the Model or S-PL groups, which indicates a near-complete restoration of community structure. Consistently, the Venn diagram (Figure 5D) reveals that the TH-PL group possessed the highest number of OTUs (945), exceeding the Model (747), S-PL (764), and Control (792) groups, with 479 OTUs common to all. The greater OTU count in the TH-PL group further demonstrates the group’s powerful effects on the gut microbiota in MS mice.

To further clarify the effects of S-PL and TH-PL supplementation, we analyzed the relative abundance of bacterial taxa at the phylum and genus levels. The gut microbiota was dominated by *Firmicutes* and *Bacteroidetes* (Figure 5E). While the Model group exhibited a marked increase in the relative abundance of *Firmicutes* compared to the Control group, administration of either S-PLs or TH-PLs effectively counteracted this change, thereby restoring the *Firmicutes* abundance to that observed in normal mice. Furthermore, supplementation with TH-PLs and S-PLs elevated the relative abundance of *Bacteroides* compared to the Model group. Analysis at the genus level revealed more distinct compositional differences. Specifically, the TH-PL group exhibited a substantial reduction in the genera *Romboutsia* and *Faecalibaculum*, alongside an increased abundance of *Odoribacter* and *Intestinimonas* (Figure 5F–I). Notably, *Odoribacter* and *Intestinimonas* are recognized as beneficial bacteria that ameliorate HFD-induced obesity and inflammation through the production of SCFAs. Therefore, we speculated that PLs might enhance the content of SCFAs by increasing the relative abundance of SCFA-producing bacteria in the gut, thus improving MS caused by HFD.

**Figure 6:**
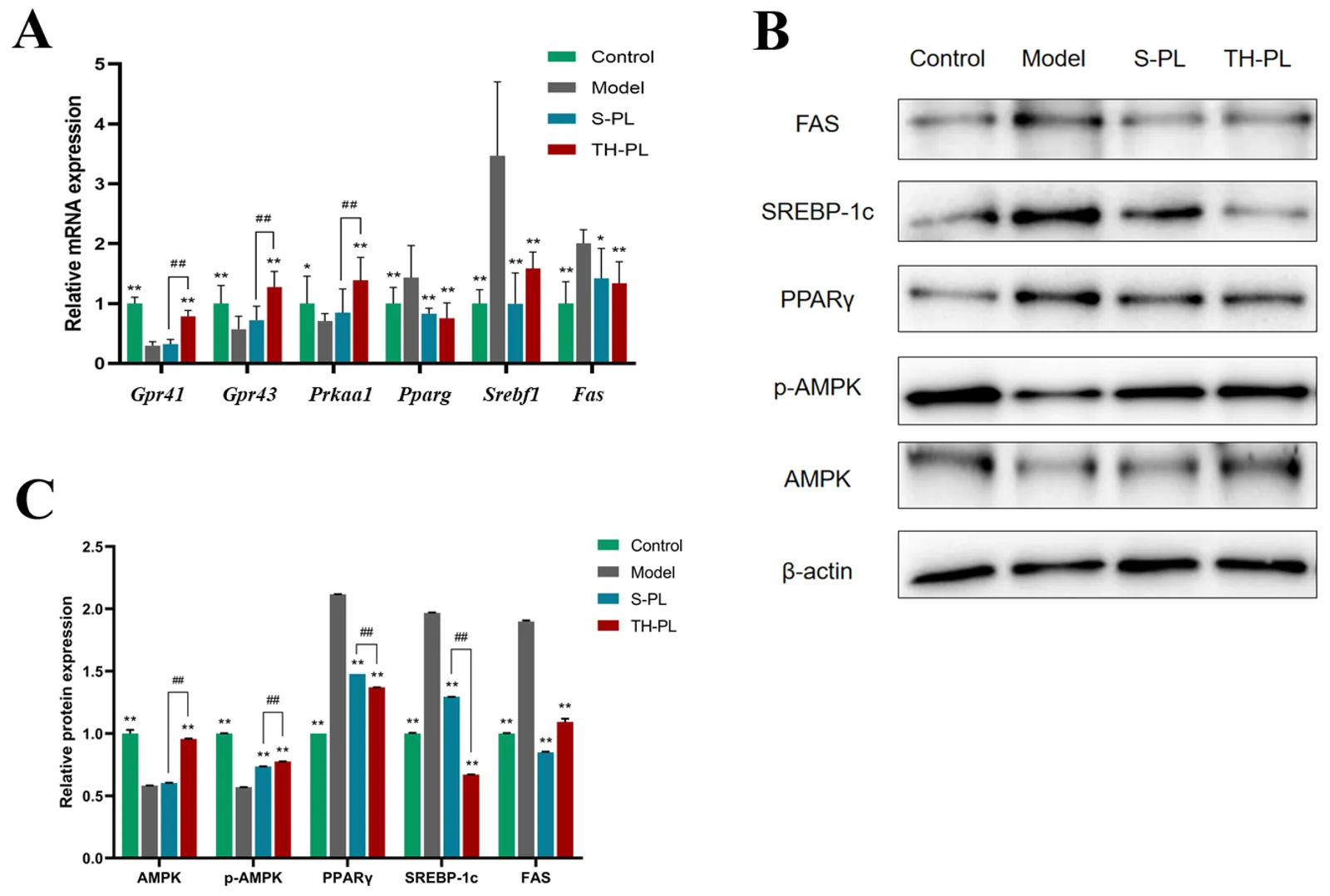
Effect of TH-PLs on liver gene and protein expression in mice. (**A**) The mRNA expression of *Gpr41*, *Gpr43*, *Prkaa1*, *Pparg*, *Srebf1* and *Fas*; (**B**) representative images of the Western blotting for AMPK, *p*-AMPK, PPARγ, SREBP-1c, and FAS in the liver, with β-actin applied as a loading control; and (**C**) the protein expression of AMPK and phosphorylation of AMPK, SREBP-1c, PPARγ and FAS. Values were expressed as mean ± SEM in each group. * *p* < 0.05 as compared to the Model group; ** *p* < 0.01 as compared to the Model group. ## *p* < 0.01 as compared to the S-PL group. (*n* = 3).

To verify this hypothesis, the levels of total and specific SCFAs in cecal contents of mice were quantified by GC (Figure 5J,K). As expected, TH-PLs significantly upregulated the total SCFA levels compared with the Model group (*p* < 0.05). Although the S-PL group showed a similar increasing trend, the difference was not statistically significant (*p* > 0.05). Specifically, the HFD significantly downregulated the levels of acetic acid, propanoic acid, butanoic acid and isovaleric acid (*p* < 0.05). By contrast, TH-PL supplementation markedly elevated the levels of propanoic, butanoic, isobutyric, valeric, and isovaleric acids (*p* < 0.05). Furthermore, the mRNA expression levels of the SCFA receptors GPR41 and GPR43 in the liver were consistent with the SCFA measurements, providing additional support for our hypothesis (Figure 6A). The expression of GPR41 and GPR43 in the liver was significantly increased by TH-PLs but not by S-PLs, correlating well with the respective SCFA levels. This indicates that the superior therapeutic effect of TH-PLs is mediated, at least in part, through a gut microbiome–SCFA–receptor axis: it enhances the abundance of SCFA-producing bacteria, thereby increasing SCFA production and potentiating receptor signaling, which is crucial for intestinal health and energy metabolism.

### 3.8. Effect of TH-PLs on Liver Gene and Protein Expression in Mice

To investigate the underlying mechanism behind TH-PL-mediated lipid metabolism regulation, we assessed the hepatic expression of key mediators (AMPK, PPARγ, SREBP-1c, and FAS) using RT-qPCR and Western blot. The Model group exhibited a profile conducive to lipogenesis, characterized by suppressed AMPK and elevated PPARγ, SREBP-1c, and FAS levels relative to the Control group. Both S-PLs and TH-PLs effectively counteracted the HFD-induced upregulation of PPARγ, SREBP-1c, and FAS. Crucially, TH-PLs uniquely and significantly enhanced AMPK expression (*p* < 0.01), an effect not seen with S-PLs. This distinct transcriptional pattern was confirmed at the protein level (Figure 6B,C), which further validates these findings.

## 4. Discussion

The pathogenesis of MS is complex, necessitating multi-target therapeutic strategies that concurrently address weight reduction, blood glucose control, dyslipidemia, IR, and gut microbiota dysbiosis. However, current common drugs typically focus on a single component of MS, which leads to drawbacks of limited efficacy and an increased risk of side effects [15]. Consequently, the development of multi-targeting natural products for the effective intervention of MS has emerged as a promising therapeutic direction. Emerging evidence indicates that *n*-3 PUFA, particularly EPA and DHA, represents a promising dietary approach for the prevention of MS [16]. Research further shows that when these fatty acids are bound to PLs rather than TG, they offer enhanced bioavailability and greater efficacy against disorders of glucose and lipid metabolism [17]. At present, EPA-/DHA-binding PLs are gaining increased attention for MS intervention. Among potential sources, fish-derived PLs present a dual advantage: they combine the bioactivities of PLs with those of *n*-3 PUFA and are available from abundant raw materials, which suggests considerable application prospects. Thus, TH-PLs were prepared and administered to C57BL/6J mice induced by HFD for 8 weeks to investigate their effects on MS in this study.

The TH-PLs were analyzed for their composition and molecular species. We identified seven PL classes and sixty-two molecular species, with PC (16:0/18:1), PE (P-16:0/22:4), PS (18:0/22:5), LPC (16:0), PI (16:0/20:4), LPE (18:0), and SM (d16:0/18:1) being the most abundant. Notably, the fatty acid composition showed a high total content of *n*-3 and *n*-6 PUFA (30.30% and 17.91%, respectively). Because aquatic PLs are a key dietary source of *n*-3 PUFA, which are primarily esterified at the sn-2 position for superior bioavailability [18,19], the high nutritional value of TH-PLs supports their potential application as a functional dietary supplement.

Throughout the establishment of the MS model, food intake and body weight were monitored. The fact that no significant differences in food intake were observed among the four groups confirmed that feed palatability did not influence the results. As expected, mice fed an HFD for 8 weeks exhibited significant increases in the Lee’s index, liver index, and epididymal fat index, which indicates the development of obesity-related phenotypes—a hallmark of MS. Although TH-PLs did not significantly reduce total body weight gain, they produced a marked decrease in these specific adiposity indices, with greater efficacy than S-PLs, which suggests a specific effect on fat distribution rather than overall energy balance.

IR, originally proposed as the core pathogenesis of MS, is characterized by a compensatory hypersecretion of insulin to maintain euglycemia, which leads to hyperinsulinemia. Furthermore, IR promotes the release of large amounts of FFAs from adipocytes, and these elevated FFAs can, in turn, exacerbate the dysfunction of pancreatic β-cells [20]. IR significantly alters cytokine expression in key metabolic tissues—including the liver, adipose, and muscle—leading to reduced cellular insulin sensitivity via secretory signaling pathways [21]. In addition, ADPN and LEP serve as vital regulators of autocrine, paracrine, and systemic insulin sensitivity [22]. Our results show that daily administration of TH-PLs or S-PLs for 8 weeks markedly improved IR in HFD-fed mice. This metabolic improvement was associated with a significant elevation in ADPN levels and reductions in FFA and LEP levels, with TH-PLs exhibiting superior efficacy. These results imply that TH-PLs effectively ameliorate the impaired insulin sensitivity in mice fed with an HFD.

Dyslipidemia represents a critical pathological state in HFD-induced MS and contributes to increased disease risk. It is prevalent in most MS patients and is clinically characterized by increased TG, TC, and LDL-C, coupled with reduced HDL-C in routine lipoprotein analysis [23]. Studies indicate that PL supplementation can ameliorate lipid deposition and enhance energy efficiency by facilitating lipid emulsification and transport, thereby reducing serum TC and LDL-C levels [24]. Furthermore, PLs can inhibit intestinal cholesterol absorption via interactions with the enterocyte membrane. In this study, while S-PLs only significantly reduced serum TC compared to the Model group, TH-PL administration markedly lowered TC, TG, and LDL-C, thereby ameliorating the overall lipid profile in HFD-induced MS mice. This superior efficacy is likely attributable to the structural specificity conferred by *n*-3 PUFA bound to PLs in TH-PLs, which appears to offer enhanced nutritional benefits. Existing studies consistently confirm that *n*-3 PUFA-bound PLs were markedly more effective than those without *n*-3 PUFA in ameliorating dyslipidemia in both plasma and the liver [9].

As the central metabolic organ, the liver is critically impaired in MS, primarily through HFD-induced lipid deposition [25]. Our histopathological assessment confirmed this pathogenesis; an HFD disrupted liver morphology, with H&E and Oil Red O staining revealing hepatic cord disarray and pronounced steatosis. Intervention with TH-PLs and S-PLs effectively mitigated this hepatic lipid accumulation. The molecular basis for this phenotypic improvement was further substantiated by the normalized expression of lipid metabolism-related genes, which points to the involvement of AMPK—a master regulator of hepatic lipid homeostasis—in the underlying mechanism. The central role of AMPK in hepatic lipid metabolism involves the coordinated regulation of catabolic and anabolic pathways. Upon activation via Thr172 phosphorylation of its α-subunit (forming *p*-AMPK) [26], AMPK not only stimulates lipid degradation but also directly suppresses de novo lipogenesis [27]. This regulatory function is critical, as evidenced by the accelerated steatosis in AMPK-deficient mice [28]. A principal mechanism by which AMPK inhibits lipogenesis is through the suppression of SREBP-1c, the master transcriptional regulator of lipogenic genes. By downregulating SREBP-1c, AMPK curtails the expression of key enzymes such as ACC and FAS—the latter being the rate-limiting step in fatty acid synthesis—thereby reducing lipid synthesis at the transcriptional level [29]. In addition, PPARγ is a transcriptional regulatory receptor that regulates adipocyte differentiation, fat metabolism, and inflammation [30]. Compared with a healthy liver, PPARγ over-expression is a general characteristic of steatosis liver, which can accelerate liver steatosis by activating lipid deposition genes and increasing triglyceride synthesis in liver [31]. Conversely, the specific knockout of liver PPARγ in mice leads to the downregulated expression of genes involved in lipogenesis (SCD1, SREBP-1c, and ACC) and lipid transport (CD36/FAT, L-FABP) [32]. Therefore, PPARγ expression in hepatocytes is a pro-steatogenic factor in fatty liver disease. PLs with different fatty acid compositions exert distinct biological effects. Studies have demonstrated that, compared with egg yolk PLs and S-PLs, DHA-PLs rich in *n*-3 PUFA more effectively inhibit SREBP-1c-mediated lipogenesis and promote hepatic lipolysis, thereby exhibiting advantages in ameliorating obesity-related metabolic disorders [10]. Furthermore, research has confirmed that krill PLs can promote the normal expression of lipid synthesis-related genes and proteins in hepatic tissue, including SREBP-1c, FAS, and PPAR-α, which highlights the considerable potential of aquatic PLs in hepatoprotection [7]. Consistent with these findings, we examined hepatic lipogenesis in the MS mouse model in the present study. The results showed that, compared with S-PLs, TH-PLs significantly upregulated the expression of AMPK and *p*-AMPK while downregulating the expression of SREBP-1c, FAS, and PPARγ. Accordingly, we determined that TH-PLs could block lipid synthesis in the liver by regulating the expression of genes involved in lipogenesis in MS mice.

Diet plays an important role in the colonization, maturation and stability of gut microbiota. A reasonable dietary structure helps to maintain the balance of gut microbiota, and a long-term HFD will not only lead to the disorder of lipid metabolism but also destroy the composition and diversity of gut microbiota, which, in turn, aggravates the metabolic disorder [33]. Thus, improving the diversity and composition of intestinal microbiota could help prevent obesity through dietary functional ingredients. This study found that after 8 weeks of an HFD, there were significant differences in the gut microbiota structure between mice in the Model group and Control group, which proved that an HFD could cause changes in the gut microbiota. S-PLs and TH-PLs could enhance the richness and diversity of gut microbiota, as manifested by increased α-diversity indexes, which contributes to their prevention of MS. Besides α-diversity, β-diversity showed significant differences among the Model and PL-administration groups, which suggests that both S-PLs and TH-PLs could regulate the composition of gut microbiota induced by an HFD. At the phylum level, S-PLs and TH-PLs could restore the relative abundance of *Firmicutes* and *Bacteroides* in MS mice compared to normal mice. PLs from egg yolk and large yellow croaker roe also showed a positive influence on HFD-induced gut dysbiosis by reducing *Firmicutes* abundance and increasing *Bacteroidetes* abundance, which is consistent with our present work [34,35]. At the genus level, TH-PLs decreased the relative abundance of *Romboutsia* and *Faecalibaculum* and increased the relative abundance of *Odoribacter* and *Intestinimonas*. *Romboutsia* exhibits a positive relationship with body weight and blood lipid levels in mice and can be used to predict metabolic diseases [36]. And *Faecalibaculum* may partly participate in the disruption of the intestinal barrier and facilitate LPS release into the blood, which can result in the aggravation of metabolic dysfunction [37]. Notably, the roles of *Intestinimonas* and *Oscillibacter* in producing SCFAs have been reported, which are considered to be beneficial genera in preventing obesity [38]. SCFAs are produced by gut microbiota for the fermentation of indigestible nutrients. They play an important role in reducing the pH of the intestinal tract, promoting the functional integrity of the intestinal barrier, inhibiting the growth of harmful bacteria in the intestine and regulating immune function [39]. The deficiency of SCFAs has been associated with metabolic diseases, and, in the current study, the level of SCFAs in the cecal contents of mice with MS was significantly reduced compared to that in normal mice. SCFAs also have the effects of inhibiting adipocyte decomposition, reducing plasma FFAs, and promoting adipocyte differentiation, and these effects may be mediated by the activation of GPR41 and GPR43 [40]. Gut microbiota may influence host lipid metabolism by producing SCFAs to stimulate GPR41 and GPR43. Acetic acid preferentially activates GPR43, butyric acid preferentially activates GPR41, and propionic acid has similar activation effects on GPR41 and GPR43 [41]. In our study, after 8 weeks of TH-PL administration, we observed increased SCFA content and elevated expression of hepatic GPR receptors, with higher concentrations than in the S-PL group. These findings raise the possibility that TH-PLs may exert more pronounced effects on MS than S-PLs, possibly through modulation of the gut microbiota and its SCFA metabolites. Collectively, our results suggest that TH-PLs might alleviate MS-related phenotypes, potentially via direct and indirect actions on HFD-induced gut microbiota disturbances, although further mechanistic studies are needed to confirm the causal relationships.

## 5. Conclusions

In this study, UPLC-Q-Orbitrap HRMS and GC-MS techniques were applied to characterize the molecular species and fatty acids composition of TH-PLs. And it was found that high levels of PUFA were distributed in the fatty acid chains of TH-PLs, which means they can be used as a potential dietary supplement for ameliorating MS. Animal experiments further revealed that administration of TH-PLs exerted more beneficial impacts on the improvement of dyslipidemia, IR and liver damage in MS mice compared with S-PLs, which was possibly associated with the effects on lipid metabolism-related gene expression, gut microbiota and its metabolites. Our study reveals the favorable anti-MS activity of TH-PLs owing to high levels of PUFA in their fatty acid chains. Furthermore, TH-PLs have demonstrated significant ameliorative effects on insulin resistance, dyslipidemia, and hepatic steatosis in the present study. TH-PLs may also hold therapeutic potential for other metabolic disorders closely associated with MS, such as non-alcoholic fatty liver disease and type 2 diabetes. Future research should encompass head-to-head comparisons with PLs derived from other aquatic sources, multi-omics mechanistic investigations, and clinical translational studies to comprehensively evaluate their broader health benefits. The development of additional TH-PL-based formula foods for the prevention and treatment of MS will be of substantial value in the future.

## Figures and Tables

**Table 1:** Primer sequences of real-time PCR.

Gene	Forward Primer	Reverse Primer
*Gpr41*	ACCACTATTTACCTCACCTCCCTCTTC	CGATGCCAGGAACCAACAGACTAC
*Gpr43*	TGCACCATCGTCATCATCGTTCAG	AGTTCTCGTAGCAGGTTATTTGGTTCTC
*Prkaa1*	TCCACAGAGATCGGGATCAGTTAGC	GAGTTAGGTCAACAGGAGAAGAGTCAAG
*Pparg*	AGCCCTTTACCACAGTTGATTTCTCC	GCAGGTTCTACTTTGATCGCACTTTG
*Srebf1*	CCTGCTTGGCTCTTCTCTTTGTCTAC	AGGTCAGCTTGTTTGCGATGTCTC
*Fasn*	GTTTAAAGCTGAGGAGGCGGGTTC	GTTTTCAGGTTGGCATGGTTGACAG
*Gapdh*	AAGAAGGTGGTGAAGCAGGCATC	CGGCATCGAAGGTGGAAGAGTG

**Table 2:** Fatty acid composition of TH-PLs.

Fatty Acids	Content (%)
C14:0	0.39 ± 0.10
C15:0	0.18 ± 0.01
C16:0	19.33 ± 2.62
C16:1	0.86 ± 0.17
C17:0	0.54 ± 0.03
C17:1	0.38 ± 0.01
C18:0	10.69 ± 0.35
C18:1	17.25 ± 1.35
C18:2n6	13.84 ± 0.29
C18:3n3	0.45 ± 0.06
C18:3n6	0.49 ± 0.05
C20:0	0.24 ± 0.02
C20:1	0.49 ± 0.08
C20:2n6	1.61 ± 0.02
C20:3n3	10.26 ± 0.70
C20:3n6	2.89 ± 0.48
C20:4n6	11.35 ± 0.78
C20:5n3 (EPA)	0.42 ± 0.04
C21:0	0.08 ± 0.01
C22:0	0.19 ± 0.01
C22:2n6	0.12 ± 0.04
C22:6n3 (DHA)	6.78 ± 0.19
C23:0	0.10 ± 0.01
C24:0	0.87 ± 0.08
C24:1	0.20 ± 0.01
∑ EPA + DHA	7.20 ± 0.15
∑ SFAs	32.59 ± 3.11
∑ MUFAs	19.20 ± 1.60
∑ *n*-3 PUFAs	17.91 ± 0.80
∑ *n*-6 PUFAs	30.30 ± 0.52

**Table 3:** Composition of major PL species in TH-PLs.

Lipid Class	Phospholipid Species	Content (%)	Total (%)
Glycerol phospholipid	PC	55.62 ± 0.42	81.76 ± 0.76
	PE	18.01 ± 0.35	
	PS	1.57 ± 0.02	
	LPC	0.39 ± 0.01	
	PI	0.23 ± 0.01	
	LPE	0.12 ± 0.01	
Sphingomyelin	SM	5.79 ± 0.09	
Other	-	18.23 ± 0.28	18.23 ± 0.28

**Table 4:** Molecular species of TH-PLs.

TClass	*m*/*z* Observed	RT (min)	Molecular Formula	Molecular Species	Relative Abundance (%)
PC	732.5521	12.64	C_40_H_78_NO_8_P	16:0/16:1	1.59 ± 0.04
	734.5692	14.06	C_40_H_80_NO_8_P	16:0/16:0	3.89 ± 0.05
	758.5687	12.99	C_42_H_80_NO_8_P	16:0/18:2	12.79 ± 0.09
	760.5843	14.21	C_42_H_82_NO_8_P	16:0/18:1	15.16 ± 0.11
	768.5883	13.77	C_44_H_82_NO_7_P	O-16:0/20:4	1.09 ± 0.01
	782.5674	11.95	C_44_H_80_NO_8_P	18:2/18:2	1.43 ± 0.01
	782.5675	12.73	C_44_H_80_NO_8_P	16:0/20:4	3.70 ± 0.02
	784.5833	13.14	C_44_H_82_NO_8_P	18:1/18:2	7.67 ± 0.04
	786.5997	14.34	C_44_H_84_NO_8_P	18:1/18:1	5.82 ± 0.02
	788.6165	15.18	C_44_H_86_NO_8_P	18:0/18:1	3.33 ± 0.01
	806.5675	12.32	C_46_H_80_NO_8_P	16:0/22:6	3.12 ± 0.01
	808.5807	12.31	C_46_H_82_NO_8_P	20:3/18:2	1.20 ± 0.01
	808.5829	12.82	C_46_H_82_NO_8_P	18:1/20:4	1.91 ± 0.01
	808.5831	13.36	C_46_H_82_NO_8_P	16:0/22:5	2.31 ± 0.02
	810.5974	14.32	C_46_H_84_NO_8_P	18:0/20:4	1.27 ± 0.01
	810.5975	13.35	C_46_H_84_NO_8_P	18:1/20:3	1.41 ± 0.02
	810.5992	13.88	C_46_H_84_NO_8_P	16:0/22:4	1.45 ± 0.01
	812.6139	14.51	C_46_H_86_NO_8_P	20:1/18:2	1.26 ± 0.01
	832.5830	12.27	C_48_H_82_NO_8_P	20:3/20:4	1.30 ± 0.01
	834.5991	12.75	C_48_H_84_NO_8_P	18:1/22:5	1.40 ± 0.02
PE	702.5419	14.31	C_43_H_78_NO_7_P	P-18:0/20:4	4.62 ± 0.05
	724.5269	12.75	C_41_H_78_NO_7_P	P-18:1/18:1	6.06 ± 0.07
	728.5586	14.40	C_45_H_78_NO_7_P	P-18:0/22:6	4.34 ± 0.05
	730.5753	15.25	C_43_H_78_NO_7_P	P-16:0/22:4	11.77 ± 0.12
	748.5258	12.31	C_43_H_76_NO_7_P	P-16:0/22:5	5.99 ± 0.06
	750.5428	13.39	C_45_H_76_NO_8_P	18:1/22:6	2.84 ± 0.04
	752.5581	13.92	C_43_H_80_NO_7_P	P-16:0/22:3	3.33 ± 0.03
	752.5630	14.38	C_43_H_76_NO_8_P	18:1/20:4	7.80 ± 0.10
	768.5535	13.64	C_45_H_80_NO_7_P	P-18:1/22:4	6.80 ± 0.13
	774.5413	12.42	C_39_H_78_NO_7_P	P-18:0/17:1	3.77 ± 0.06
	776.5575	13.95	C_39_H_74_NO_7_P	P-16:0/18:2	3.51 ± 0.02
	778.5737	14.78	C_45_H_84_NO_7_P	O-18:1/22:3	2.90 ± 0.02
	780.5906	15.04	C_41_H_76_NO_7_P	P-18:1/18:2	4.16 ± 0.03
	790.5363	11.62	C_41_H_76_NO_7_P	P-18:0/18:3	2.02 ± 0.02
	794.5671	14.25	C_47_H_78_NO_8_P	22:4/20:4	3.78 ± 0.01
PS	788.5434	9.96	C_42_H_78_NO_10_P	18:1/18:1	9.00 ± 0.05
	790.5595	10.54	C_42_H_80_NO_10_P	18:0/18:1	12.27 ± 0.09
	812.5431	9.89	C_44_H_78_NO_10_P	18:0/20:4	9.92 ± 0.11
	814.5589	10.15	C_44_H_80_NO_10_P	18:0/20:3	5.86 ± 0.06
	836.5429	9.70	C_46_H_78_NO_10_P	18:0/22:6	14.66 ± 0.14
	838.5587	10.17	C_46_H_80_NO_10_P	18:0/22:5	24.20 ± 0.28
	840.5737	10.40	C_46_H_82_NO_10_P	18:0/22:4	19.63 ± 0.17
LPC	482.3607	5.29	C_24_H_52_NO_6_P	O-16:0	6.87 ± 0.09
	496.3404	4.43	C_24_H_50_NO_7_P	16:0	33.83 ± 0.20
	520.3400	3.05	C_26_H_50_NO_7_P	18:2	13.01 ± 0.17
	522.3559	5.06	C_26_H_52_NO_7_P	18:1	23.62 ± 0.26
	524.3718	6.81	C_26_H_54_NO_7_P	18:0	11.56 ± 0.07
PI	876.5594	8.87	C_45_H_79_O_13_P	16:0/20:4	66.28 ± 0.15
	902.5749	8.95	C_47_H_81_O_13_P	18:1/20:4	19.61 ± 0.07
	904.5908	9.58	C_47_H_83_O_13_P	18:0/20:4	5.31 ± 0.01
	906.6061	9.82	C_47_H_85_O_13_P	18:0/20:3	4.69 ± 0.02
	928.5912	9.42	C_49_H_83_O_13_P	18:1/22:5	4.07 ± 0.02
LPE	482.3246	5.37	C_23_H_48_NO_7_P	18:0	50.66 ± 0.15
	502.2936	2.22	C_25_H_44_NO_7_P	20:4	26.87 ± 0.08
	526.2938	2.13	C_27_H_44_NO_7_P	22:6	22.45 ± 0.06
SM	703.5738	12.43	C_39_H_79_N_2_O_6_P	d16:0/18:1	19.17 ± 0.21
	759.6393	15.21	C_43_H_87_N_2_O_6_P	d20:0/18:1	4.67 ± 0.06
	785.6534	15.14	C_45_H_89_N_2_O_6_P	d16:0/24:2	9.01 ± 0.05
	787.6702	15.98	C_45_H_91_N_2_O_6_P	d16:0/24:1	10.97 ± 0.02
	811.6687	15.25	C_47_H_91_N_2_O_6_P	d18:2/24:1	10.25 ± 0.03
	813.6855	15.90	C_47_H_93_N_2_O_6_P	d18:1/24:1	17.97 ± 0.11
	815.7014	16.89	C_47_H_95_N_2_O_6_P	d16:0/26:1	5.60 ± 0.04

## Data Availability

The original contributions presented in this study are included in the article. Further inquiries can be directed to the corresponding author.

## Abbreviations

The following abbreviations are used in this manuscript:

PL	phospholipid
TH-PL	tilapia head phospholipid
S-PL	soybean phospholipid
PC	phosphatidylcholine
PE	phosphatidylethanolamine
PS	phosphatidylserine
PI	phosphatidylinositol
LPC	lysophosphatidylcholine
LPE	lysophosphatidyleth-anolamine
PA	phosphatidic acid
SM	sphingomyelin
SFA	saturated
PUFA	polyunsaturated
MUFA	monounsaturated
EPA	eicosapentaenoic acid
DHA	docosahexaenoic acid
MS	metabolic syndrome
HFD	high-fat diet
TG	Triglycerides
TC	Total cholesterol
HDL-C	High-density lipoprotein cholesterol
LDL-C	Low-density lipoprotein cholesterol
ALT	Alanine Aminotransferase
AST	Aspartate Aminotransferase
ADPN	Adiponectin
LEP	Leptin
FFA	free fatty acid
SCFA	short-chain fatty acid
